# Intranasal Administration of Maleic Anhydride-Modified Human Serum Albumin for Pre-Exposure Prophylaxis of Respiratory Syncytial Virus Infection

**DOI:** 10.3390/v7020798

**Published:** 2015-02-16

**Authors:** Zhiwu Sun, Qian Wang, Ran Jia, Shuai Xia, Yuan Li, Qi Liu, Wei Xu, Jin Xu, Lanying Du, Lu Lu, Shibo Jiang

**Affiliations:** 1 Key Lab of Medical Molecular Virology of MOE/MOH, Shanghai Medical College, Fudan University, 130 Dong An Rd., Xuhui District, Shanghai 200032, China; E-Mails: haoyunzhiwu@163.com (Z.S.); miaqian@foxmail.com (Q.W.); 928958768@qq.com (S.X.); liyuan.mic@gmail.com (Y.L.); qiliu@aliyun.com (Q.L.); xuwei0576@126.com (W.X.); 2 Laboratory Medicine Center, Pediatric Institute, Children’s Hospital of Fudan University, 399 Wanyuan Road, Shanghai, 201102, China; E-Mails: goodbye_sea@126.com (R.J.); jinxu_125@163.com (J.X.); 3 Lindsley F. Kimball Research Institute, New York Blood Center, New York, NY 10065, USA; E-Mail: lanydu2007@gmail.com (L.D.)

**Keywords:** human serum albumin (HSA), pre-exposure prophylaxis (PrEP), respiratory syncytial virus (RSV), entry inhibitor, antiviral

## Abstract

Respiratory syncytial virus (RSV) is the leading cause of pediatric viral respiratory tract infections. Neither vaccine nor effective antiviral therapy is available to prevent and treat RSV infection. Palivizumab, a humanized monoclonal antibody, is the only product approved to prevent serious RSV infection, but its high cost is prohibitive in low-income countries. Here, we aimed to identify an effective, safe, and affordable antiviral agent for pre-exposure prophylaxis (PrEP) of RSV infection in children at high risk. We found that maleic anhydride (ML)-modified human serum albumin (HSA), designated ML-HSA, exhibited potent antiviral activity against RSV and that the percentages of the modified lysines and arginies in ML- are correlated with such anti-RSV activity. ML-HSA inhibited RSV entry and replication by interacting with viral G protein and blocking RSV attachment to the target cells, while ML-HAS neither bound to F protein, nor inhibited F protein-mediated membrane fusion. Intranasal administration of ML-HSA before RSV infection resulted in significant decrease of the viral titers in the lungs of mice. ML-HSA shows promise for further development into an effective, safe, affordable, and easy-to-use intranasal regimen for pre-exposure prophylaxis of RSV infection in children at high risk in both low- and high-income countries.

## 1. Introduction

Human respiratory syncytial virus (RSV), a member of the paramyxoviridae family of negative-sense RNA viruses, is the leading cause of virus-induced severe lower respiratory tract disease in infants and children as well as morbidity and mortality in elderly people and immunocompromised adults [1,2,3]. An estimated 33.8 million cases of acute lower respiratory infection (ALRI) caused by RSV infection in children younger than 5 years occurred in 2005, and about 66,000–199,000 of these infected children died from ALRI [4]. In the United States, about 125,000 hospitalizations and 2.1 million children under 5 years of age require medical attention each year because of RSV infection [5]. Virtually every child has been infected by RSV at any given age [6,7].

Neither vaccine nor effective antiviral therapy is currently available to prevent and treat RSV infection. Palivizumab (trade name: Synagis) is a humanized murine monoclonal antibody (mAb) specific for RSV F glycoprotein with neutralizing and fusion inhibitory activity against RSV. It has been approved by the U.S. Food and Drug Administration (FDA) as passive immunoprophylaxis for use among infants at high risk, including those born prematurely and those with chronic lung disease or congenital heart disease [8,9,10]. However, its high cost ($2,962 per 100-mg vial of palivizumab in June 2013) prevents its use in low-income countries [11], and as a mAb, it has to be maintained and transported under low temperature. Furthermore, palivizumab must be prescribed by pediatricians or physicians and intramuscularly injected at doctors’ offices, hospitals, or clinics, making its use inconvenient. An ideal prophylactic RSV drug should be effective, safe, affordable and easy to use for both infants and the elderly. Therefore, we aimed to identify an antiviral agent that is expected to be as effective and safe as palivizumab, but more affordable and easier to use for pre-exposure prophylaxis (PrEP) of RSV infection among infants at high risk.

Our previous studies have demonstrated that anhydride-modified proteins exhibit potent antiviral activities against human immunodeficiency virus 1 (HIV-1), herpes simplex virus (HSV) and human papillomavirus (HPV) [12,13,14]. Here, we found that anhydride-modified proteins, particularly 3-hydroxyphthalic anhydride (HP) and maleic anhydride (ML) modified bovine albumin (BSA) and human serum albumin (HSA), exhibited potent inhibitory activities against RSV. We selected ML-modified HSA (ML-HSA) for further study because ML is much cheaper than HP, while HSA is much safer than BSA for human use. The mechanism study indicated that ML-HSA inhibits RSV entry and replication by blocking RSV attachment to the target cells though its interaction with viral G protein. Intranasal administration of ML-HSA before RSV challenge led to significant decrease of viral titers in the lungs of mice, suggesting that ML-HSA may be used for PrEP of RSV infection in children at high risk.

## 2. Materials and Methods

### 2.1. Reagents

3-hydroxyphthalic anhydride (HP), maleic anhydride (ML), succinic anhydride (SU), human serum albumin (HSA), chicken ovalbumin (OVA), bovine serum albumin (BSA), and β-lactoglobulin (β-LG) were purchased from Sigma (St. Louis, MO, USA). Cell Counting Kit-8 (CCK-8) was bought from Dojindo Molecular Technologies, Inc. (Dojindo, Japan). 2,4,6-trinitrobenzenesulfonic acid (TNBS) was purchased from Sigma. ρ-hydroxyphenylglyoxal (ρ-HPG) was purchased from Fisher Scientific Co. (Valley Park, VA, USA). BMS433771 and TMC353121 were purchased from Sigma. Anti-RSV antibody (NCL-RSV3) was from Leica Biosystems (Leica Biosystems Newcastle Ltd., Newcastle Upon Tyne, UK). Rabbit anti-mouse HRP antibody was purchased from Dako (Dako, Glostrup, Denmark). Low melting point agarose was bought from Invitrogen (Invitrogen, Carlsbad, CA, USA).

### 2.2. Cells, Virus and Plasmid

HEp-2 cells and Vero cells were obtained from American Type Culture Collection (ATCC). Flp-In 293 cells were obtained from Invitrogen. They were maintained in Eagle’s minimal essential medium (DMEM) (Gibco/BRL, Grand Island, NY, USA), supplemented with 5% fetal bovine serum (FBS) from Gibco, 2 mM L-glutamine, 100 U of penicillin and 100 ng of streptomycin per mL. RSV Long Strain was purchased from ATCC and grown in HEp-2 cells. The pcDNA5/FRT/TO and pOG44 vectors were purchased from Invitrogen. The pUC57-F vector encoding the F protein of RSV A2 was provided by Dr. Bin Wang at Fudan University.

### 2.3. Determination of RSV Titer

Viral titer was determined by plaque assay as previously described [15]. Briefly, serial 10-fold dilutions of RSV Long Strain/A2 strain in DMEM with 2% FBS were used to infect HEp-2 monolayers on 24-well plates at 1 × 10^5^ cells per well in duplicates for 3 h at 37 °C. After virus attachment to cells, the inocula were removed and washed with phosphate buffer solution (PBS). The infected cells were overlaid with 2% FBS in DMEM containing 0.5% low melting point agarose and incubated at 37 °C. Five days later, plaques were developed, and cells were fixed with 4% paraformaldehyde. After removal of agarose, viral plaques were visualized by immunoperoxidase staining with mouse anti-RSV (NCL-RSV3) antibody as the primary antibody (1:500 in PBS) and HRP-conjugated rabbit anti-mouse IgG as the secondary antibody (1:250 dilution in PBS). HRP Color Development Reagent (4CN, Bio-Rad, Hercules, CA, USA) was used for color development of plaques.

### 2.4. Chemical Modification of Proteins with Different Anhydrides under Variable Conditions

Protein modification was conducted as previously described [13]. Briefly, the proteins were dissolved in 0.1 M phosphate (final concentration, 20 mg/mL). Subsequently, 3-hydroxyphthalic anhydride (HP), maleic anhydride (ML) or succinic anhydride (SU) was added, respectively, in five aliquots with a 12-min interval, while pH was adjusted to 9.0. To optimize the condition of anhydride modification, HSA was modified with 2.5, 5, 10, 20, 40 or 60 mM anhydride (HP, ML or SU), respectively. The mixtures were kept for another 1 h at room temperature and then extensively dialyzed against PBS.

Protein concentrations were determined with the Pierce BCA Protein Assay Kit (Thermo, Rockford, IL, USA). To determine lysine residues in modified or unmodified proteins, TNBS assay was performed as previously described [16,17]. Briefly, 25 μL Na_2_B_4_O_7_ (0.1 M, pH 8.5) were added to 25 μL modified or unmodified proteins for 5 min at room temperature. Then, 3 μL of TNBS and 7 μL of PBS were added to the mixture. After 1 h, a 100-μL stop solution (0.1 M NaH_2_PO_4_ and 1.5 mM Na_2_SO_3_) was added to terminate the reaction. The absorbance at 420 nm (A_420_) was measured with a microplate reader (Infinite M200 Pro, Tecan, Research Triangle Park, NC, USA). The assay utilized to measure the percentage of arginine residue modification was performed as previously described [18,19]. ρ-HPG (10 μL, 50 mM) was added to 90 μL anhydride-modified or unmodified proteins dissolved in 0.1 M sodium phosphate (pH 9.0) and kept at room temperature in the dark for 90 min. The absorbance at 340 nm (A_340_) was measured.

### 2.5. Cytotoxicity Assay

The *in vitro* cytotoxicity of the anhydride-modified and unmodified HSA to the target cells used for measuring RSV infectivity (HEp-2 and Vero) was measured with a CCK-8 kit, according to the manufacturer’s instructions [20]. Briefly, 100 μL of modified and unmodified proteins at graded concentrations were added to equal volumes of cells (4 × 10^4^/mL) in wells of 96-well plates. After incubation at 37 °C for 4 days, 10 μL of CCK-8 solution were added. After 4 h of incubation, the absorbance at 450 nm (A_450_) was determined with an ELISA reader (Infinite M200 Pro).

### 2.6. Assay for Cell Protection of Anhydride-Modified Proteins against RSV

An assay for cell protection, as described previously [21], was used to assess the antiviral activities of anhydride-modified proteins. In brief, HEp-2 cells were seeded into a 96-well plate at 4000 cells per well; then serially diluted proteins were added to the plated HEp-2 cells and infected the with 4.0 × 10^2^ plaque-forming unit (PFU) of RSV Long Strain (MOI = 0.1). After culture at 37 °C for five days, the cell viability was examined by CCK-8 kit as described above.

### 2.7. Time-of-Addition and Temperature Shift Assays

To investigate the mechanism of action of ML-HSA against RSV, time-of-addition and temperature shift assays were performed as previously described [22,23,24]. Monolayer cultures of HEp-2 cells were infected with 2 × 10^5^ PFU (MOI = 2) of RSV Long Strain in the absence or presence of ML-HSA (final concentration, 2000 nM). ML-HSA was added to the plates at 0, 0.5, 1, 2, 3, 5, or 7 h post-infection. At 20 h post-infection, supernatants were collected, and inhibition of RSV infection was determined by plaque assay as described above. In temperature shift assays, HEp-2 cells were plated as described above and exposed to RSV Long Strain at 4 °C in the presence of various amounts of ML-HSA. Heparin, an RSV attachment inhibitor [24], was included as a control. After 1 h of incubation, cells were washed with ice-cold PBS twice and replaced with fresh medium. As a control, cells in the presence of ML-HSA or heparin were not washed. The plates were then moved to an incubator at 37 °C. After culture at 37 °C for 5 days, the cytopathic effect (CPE) was determined with CCK-8 kit as described in the previous section.

### 2.8. Cell-Cell Fusion Assay

To investigate whether ML-HSA could inhibit RSV F protein-mediated cell-cell fusion or syncytium formation, we performed a cell-cell fusion assay based on the fact that RSV F protein expressed on the cell surface can mediate cell fusion with neighboring cells [2,25]. To construct the 293-F cells expressing F protein of RSV, F gene of RSV A2 fused with GFP at its C-terminus was cloned into pcDNA5/FRT/TO vector (pcDNA5/FRT/TO-F). Then pcDNA5/FRT/TO-F and pOG44 were co-transfected into the Flp-In 293 cells with a 1:9 ratio. After 48 h of transfection, cells were split and added with 200 μg/mL Zeocin (Invitrogen) and 100 μg/mL Hydromycin B (Invitrogen). Then, 2 × 10^5^ 293-F cells per well were seeded at 24-well plate. After incubation at 37 °C for 24 h, 2 μg/mL tetracycline and 1% DMSO was added to induce the F protein expression on the 293-F cells. ML-HSA (1000 nM), HSA (1000 nM), and TMC353121 (200 nM, an F protein inhibitor) [26], were added to the 293-F cells. After coculture for 48 h, the cell-cell fusion or syncytium formation was visualized by microscopy (Nikon, Eclipse TS 100, Nikon, Tokyo, Japan).

### 2.9. ELISA Assay

To determine the interaction between ML-HSA and RSV G protein, we performed an ELISA assay as previously described [27]. Briefly, RSV G protein (extracellular domain, provided by Dr. B. Wang at Fudan University) at 5 μg/mL was coated on 96-well plates and incubated overnight at 4 °C. Plates were blocked with 1% gelatin in PBS for 1 h at 37 °C, followed by incubation with HSA or ML-HSA serially diluted in PBS for 30 min at 37 °C. Anti-HSA antibody (Abcam, Hong Kong) was added at 1.5 μg/mL and incubated for 1 h at 37 °C. Horseradish peroxidase (HRP)-conjugated goat anti-human IgG antibody (Dako, Glostrup, Denmark) at 1:2500 dilution was added and incubated at 37 °C for 1 h. Plates were washed between each step with 0.05% Tween 20 in PBS. Plates were developed using 3,3’,5,5’-tetramethylbenzidine (TMB) peroxidase substrate (Sigma), and absorbance at 450 nm (A_450_) was measured using an ELISA reader (Infinite M200 Pro). The interaction between ML-HSA and RSV F protein was also determined by ELISA described above. F protein was purchased from Sino Biological Inc. (Beijing, China). In the control assay, 10 μg/mL each of HSA and ML-HSA were coated on 96-well plates, and ELISA assay was performed as described above.

### 2.10. Selection of Drug-Resistant Virus

Drug-resistant RSV strains were selected and purified as previously described [28,29]. Briefly, 8 × 10^5^ HEp-2 cells were seeded on 6-well plates (Corning, Acton, MA, USA). Cells were infected with RSV Long Strain (wild-type strain) at an MOI > 1 in the presence or absence of ML-HSA at increasing concentration (starting at 63 nM and gradually increasing to 3690 nM). The virus was then passaged continuously until it showed resistance to ML-HSA. The wild-type strain was passaged without drugs in parallel. Following 8 passages of the RSV Long Strain in the presence of ML-HSA, the first resistant strain was obtained (IC_50_ = 8.39 nM). At passage 15 under ML-HSA pressure, the second resistant virus strain (IC_50_ = 201 nM) was obtained and used as the resistant strain for sequence analysis.

### 2.11. RSV-RNA Extraction and RT-PCR Amplification

Viral RNA was extracted from supernatant of the RSV-infected HEp-2 cells using a commercial kit (High Pure Viral Nucleic Acid kit, Roche Diagnostics, Indianapolis, IN, USA). For gene amplification, primers were used as described in previous studies [28]. The specific G primers were used to perform the reverse transcription of viral RNA to acquire the specific cDNA of the G genes. Briefly, 2 μL each of extracted RNA and 1 μL of primer were combined with 0.5 mM dNTP mixture, first-strand buffer, 10 μM DTT, and 40 units of SuperScript^®^ IIRNase H^-^ transcriptase (Invitrogen) in 20 μL of reaction volume. The mixture was incubated at 42 °C for 50 min and followed by heating at 70 °C for 15 min. RSV G protein genes were amplified from the specific cDNAs gained as above by nested PCR using PrimeSTAR^®^ HS DNA Polymerase (TakaRa, Japan) and the primer sets and cycle parameters as described in previous research [28]. PCR products from the second round were, as expected, 1088 base pairs (bp), and they were visualized by electrophoresis on a 0.8% agarose gel stained with ethidium bromide.

### 2.12. Mouse Models of RSV Infection

Female BALB/c mice six to eight weeks of age were purchased from the Animal Center of Fudan University (Shanghai, China). Mice were housed in cages with barrier filters in certified rooms and were fed and watered ad libitum. The protocol for the use of animals in this study was approved by the Animal Center of Fudan University (Permit number: 2013N-067). The animal studies were conducted in strict accordance with the recommendations in the Guide for the Care and Use of Laboratory Animals.

A mouse model of RSV infection was used to examine the *in vivo* inhibitory activity of ML-HSA as described previously [30,31,32]. Briefly, BALB/c mice were chemically immunosuppressed by intraperitoneal injection of 100 mg of cyclophosphamide (Sigma) per kg of body weight 6 days and 1 day prior to RSV infection. The mice were intranasally administered with different doses of ML-HSA mixed with 2.1 × 10^5^ PFU RSV. Four days after RSV infection, the mice were sacrificed. Lung homogenates (10%; wt/vol) were prepared in Hanks balanced salt solution containing 0.21 M sucrose, 25 mM HEPES, and 5 mM sodium L-glutamate supplemented with 20 U/mL of penicillin G, 20 μg/mL of streptomycin, and 0.05 μg/mL of amphotericin B (Sigma). Lung homogenates were frozen on dry ice and thawed to release cell-associated virus. Samples of lung homogenates were centrifuged at 300 × *g* for 10 min at 4 °C, and the supernatants were collected for viral titration in HEp-2 cells. In brief, each test sample was assayed in duplicate sets of serial 3.17-fold dilutions in serum-free DMEM supplemented with 2 mM L-glutamine, 100 U of penicillin and 100 μg of streptomycin per mL. Each 200-μL diluted sample was plated to HEp-2 cells in flat-bottom 24-well polystyrene plates (Corning, Acton, MA, USA). Then plaque assay was performed as described above.

To evaluate the prophylactic effect of ML-HSA, mice were intranasally administered with ML-HSA at different concentration. About 30 min or 15 min later, the mice were intranasally inoculated with 2.1 × 10^5^ PFU of RSV, respectively. After 4 days, the mice were sacrificed, and the viral titer in the lung homogenates was determined as described above.

## 3. Results

### 3.1. Anhydride-Modified Proteins were Potent Inhibitors against RSV Infection

Previous studies have demonstrated that bovine milk proteins modified with anhydride become highly potent inhibitors against HIV-1 and HPV infection [12,13]. With a similar approach, we modified four proteins, including β-LG, BSA, OVA and HSA, with HP, ML and SU, respectively. Their antiviral activities against infections of RSV A2 and Long Strain were tested. As shown in Table 1, except HP that showed moderate inhibitory activity at high concentration, ML and SU exhibited no inhibition at the concentration up to 10 µM. However, all of these modified proteins exhibited highly effective antiviral activities against both A2 and Long strains with IC_50_s at the nanomolar level. The anhydride-modified BSA and HSA were more efficacious than the other two anhydride-modified proteins, while the RSV A2 strain was more sensitive than the RSV Long Strain to the anhydride-modified proteins. Although anhydride-modified BSA exhibited anti-RSV activity similar to anhydride-modified HSA, we selected anhydride-modified HSA for further studies because some infants may be allergic to BSA, a bovine protein, but not HSA, a human protein.

**Table 1:** Comparison of the anti-respiratory syncytial virus (RSV) activities of different proteins modified by distinct anhydrides.

Anhydride-Modified Protein	Inhibition of Infection by			
	RSV A2 Strain		RSV Long Strain	
	IC_50_ (μM)	IC_90_ (μM)	IC_50_ (μM)	IC_90_ (μM)
HP	3.142 ± 0.458	>10	5.832 ± 0.753	>10
HP-β-LG	0.062 ± 0.011	0.227 ± 0.029	0.153 ± 0.011	0.422 ± 0.021
HP-OVA	0.013 ± 0.002	0.052 ± 0.009	0.100 ± 0.007	0.517 ± 0.121
HP-HSA	0.006 ± 0.001	0.042 ± 0.022	0.011 ± 0.003	0.056 ± 0.010
HP-BSA	0.005 ± 0.001	0.025 ± 0.010	0.016 ± 0.004	0.099 ± 0.011
ML	>10	>10	>10	>10
ML-β-LG	0.283 ± 0.130	1.046 ± 0.263	0.173 ± 0.045	0.581 ± 0.091
ML-OVA	0.024 ± 0.011	0.077 ± 0.033	0.217 ± 0.005	1.425 ± 0.143
ML-HSA	0.012 ± 0.002	0.038 ± 0.003	0.002 ± 0.001	0.013 ± 0.005
ML-BSA	0.002 ± 0.000	0.026 ± 0.019	0.007 ± 0.002	0.025 ± 0.005
SU	>10	>10	>10	>10
SU-β-LG	0.599 ± 0.079	1.388 ± 0.112	0.405 ± 0.100	1.093 ± 0.209
SU-OVA	0.046 ± 0.008	0.172 ± 0.024	0.277 ± 0.077	1.173 ± 0.321
SU-HSA	0.011 ± 0.005	0.049 ± 0.021	0.014 ± 0.005	0.110 ± 0.070
SU-BSA	0.006 ± 0.001	0.047 ± 0.011	0.026 ± 0.015	0.125 ± 0.020

Each sample was tested in triplicate, and the experiment was repeated twice. The data from one representative experiment were presented in mean ± SD.

**Figure 1:**
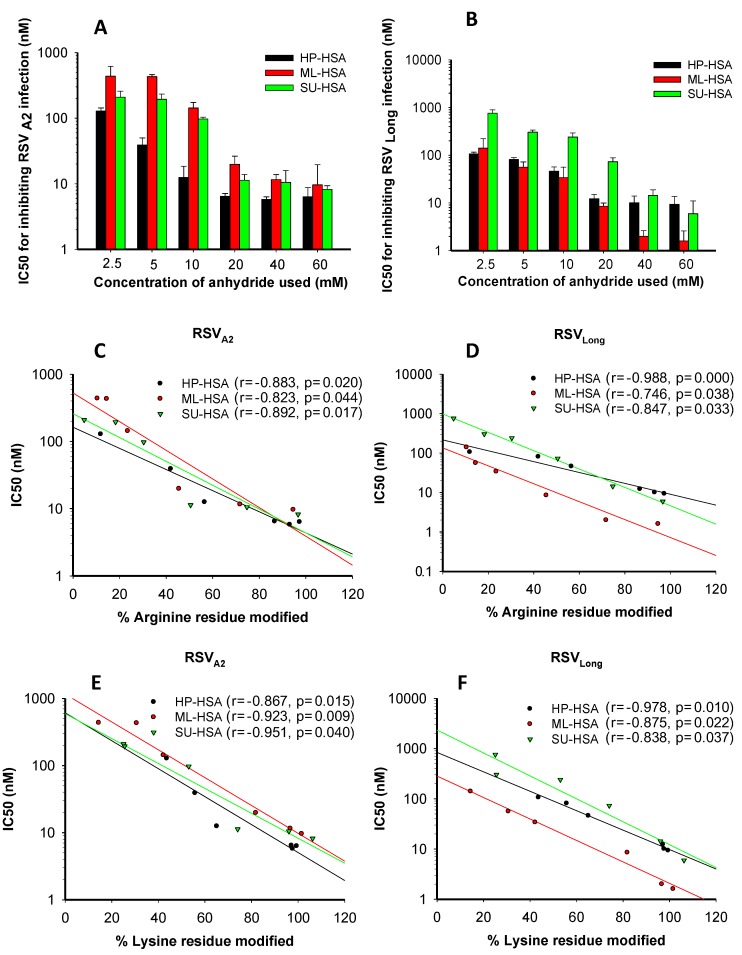
The effects of anhydride concentrations in the reaction system on anti-RSV activity of the HP-, ML-, and SU-modified human serum albumin (HSA) and the correlation between the percentages of modified residues and anti-RSV activity of the modified HSA. The IC_50_ values of HSA modified with HP, ML, and SU, respectively, for inhibiting infection by RSV A2 Strain (**A**) and Long Strain (**B**). The correlation between the percentages of the modified arginine residues and the antiviral activities of the modified HSA against RSV A2 Strain (**C**) and Long Strain (**D**). The correlation between the percentages of lysine residues and the antiviral activities of the modified HSA against RSV A2 strain (**E**) and Long Strain (**F**). Each sample was tested in triplicate, and the experiment was repeated twice. The data from one representative experiment were presented in mean ± SD.

**Figure 2:**
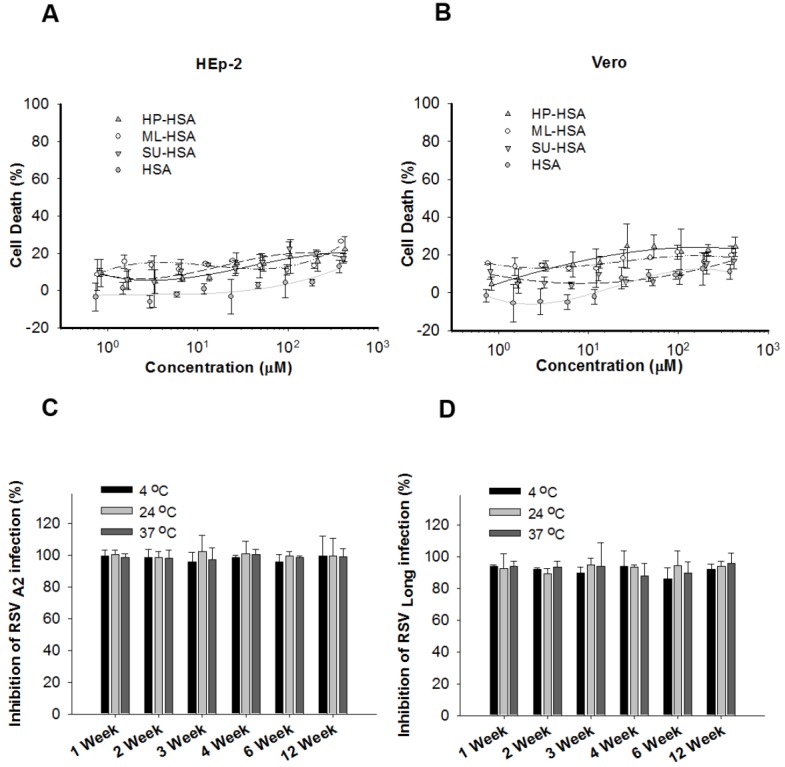
Cytotoxicity of different anhydride-modified HSA and the stability of ML-HSA. Cytotoxicity of HSA modified with HP, ML, and SU, respectively, to HEp-2 cells (**A**) and Vero cells (**B**) was determined by CCK8 kit. ML-HSA was stored at 4, 24 and 37 °C, respectively, for one to twelve weeks, followed by assessment of its antiviral activities against infection by RSV A2 strain (**C**) and Long Strain (**D**), respectively, were detected at the indicated time. Each sample was tested in triplicate, and the experiment was repeated twice. The data from one representative experiment were presented in mean ± SD.

### 3.2. The Percentages of Modified Residues of arginine and Lysine Correlate with Anti-RSV Activity of Anhydride-Modified Proteins

Previous studies have shown that the percentages of modified arginine and lysine residues were associated with the concentration of anhydrides used [19]. To identify an anhydride with optimal concentration for producing the most effective anti-RSV agent, we used SU, ML and HP at different concentrations to modify HSA and calculated the percentages of modified arginine and lysine residues, as well as tested their antiviral activity against RSV A2 Strain and Long Strain. As shown in Figure 1A,B, with the increasing concentrations of anhydrides used, the percentages of the modified arginine and lysine residues increased, and the modified proteins gained more potent anti-RSV activity. We observed a highly significant direct correlation between the percentages of the modified arginine residues and the inhibitory activity against RSV A2 and Long strains (Figure 1C,D). For ML-HSA against RSV A2 and RSV Long strains, *r* = −0.823; *p* = 0.044 and *r* = −0.746; *p* = 0.038, respectively. The percentages of modified lysine residues also directly correlated with the anti-RSV activity of anhydride-modified proteins (Figure 1E,F). For ML-HSA against RSV A2 and RSV Long strains, *r* = −0.923; *p* = 0.009 and *r* = −0.875; *p* = 0.022, respectively. Neither anhydride-modified HSA nor unmodified HSA showed significant cytotoxicity at concentrations as high as 400 µM (Figure 2A,B). Based on the results from our previous studies [19,32] and the present study, we selected the average pH of 9.0 and 60 mM of anhydride as the optimal parameters for subsequent experiments.

SU-modified HSA was less efficacious against infections of RSV A2 and Long Strain than HP- and ML-modified HSA. We thus excluded SU-HSA from subsequent study. Although HP-HSA had a similar, or even better, anti-RSV activity than ML-HSA, the cost of HP is much higher than that of ML. We thus selected ML-HSA for further studies. To study stability of ML-HSA, we kept ML-HSA at 4, 24, and 37 °C, respectively, for 1 to 12 weeks, and detected its anti-RSV activities at the week 1, 2, 3, 4, 6, and 12, respectively. Since its anti-RSV activity exhibited no significant changes during the 12-week storage period, ML-HSA was determined highly stable (Figure 2C,D).

### 3.3. Time-of-Addition and Temperature Shift Studies Suggest that ML-HSA Inhibits RSV Infection by Blocking RSV Attachment to the Target Cells

**Figure 3:**
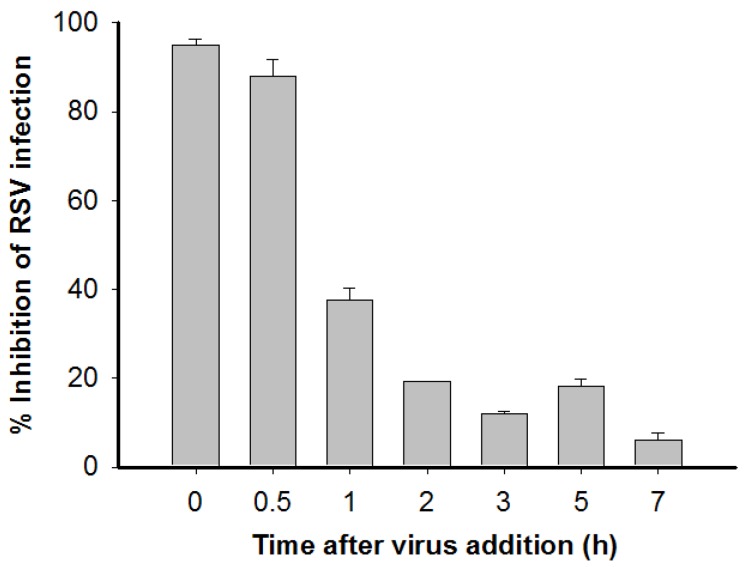
HSA-ML blocked RSV entry into the target cell. A time-of-addition assay was performed to determine the mechanism of action of ML-HSA. Each sample was tested in triplicate, and the experiments were repeated twice. The data from one representative experiment were presented in mean ± SD.

To determine the step of virus replication at which ML-HSA exerts its antiviral effect, ML-HSA was added at different times after RSV infection. Supernatants were then collected to determine viral infectivity at 20 h post-infection. As shown in Figure 3, ML-HSA was effective in inhibiting RSV replication (95%) if added at the same time as virus inoculation. When ML-HSA was added 0.5 h after infection, it still displayed a powerful inhibitory activity (88%). However, when added to the cell culture 1 h post-infection, the percentage of inhibition of ML-HSA was reduced to 38%. When ML-HSA was added 2 to 7 h post-infection, no significant inhibition of viral infection was seen. These results suggest that ML-HSA inhibits RSV infection by targeting the early step of virus replication, which is viral entry.

**Figure 4:**
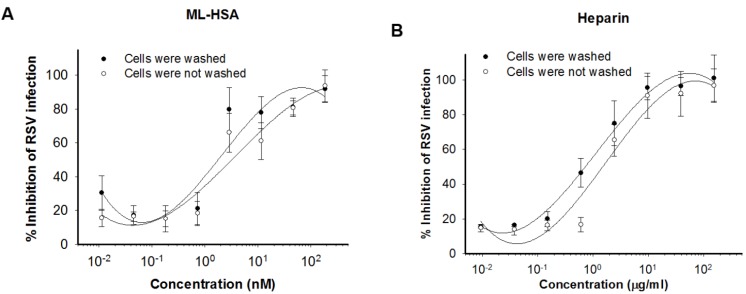
ML-HSA blocked RSV attachment. HEp-2 cells were incubated with RSV in the presence of serial dilutions of either ML-HSA (**A**) or heparin (**B**) at 4 °C for 1 h, followed by washes to remove unbound virus and inhibitor, or no washing before the temperature was shifted to 37 °C. CPE was determined after 5 days and plotted as cell viability. Each diluted sample was tested in triplicate, and the experiment was repeated twice. The data from one representative experiment were presented in mean ± SD.

To further explore the mechanism of action of ML-HSA, we performed a temperature shift assay based on a report that RSV could attach to cells at 4 °C, while its fusion with the target cell membrane occurs at temperatures above 18 °C [33]. Thus, a viral attachment inhibitor can be expected to maintain antiviral activity if it is added to the mixture of cells and virus at 4 °C, followed by washing before incubation temperature is shifted to 37 °C. As shown in Figure 4A, ML-HSA exhibited similar anti-RSV activity whether the cells were washed, or not, after it was added to the mixture of cells and virus at 4 °C. Heparin, a known RSV attachment inhibitor, also showed similar anti-RSV potency whether the cells were washed or not (Figure 4B). These data indicate that ML-HSA inhibits RSV infection by blocking virus attachment to cells.

**Figure 5:**
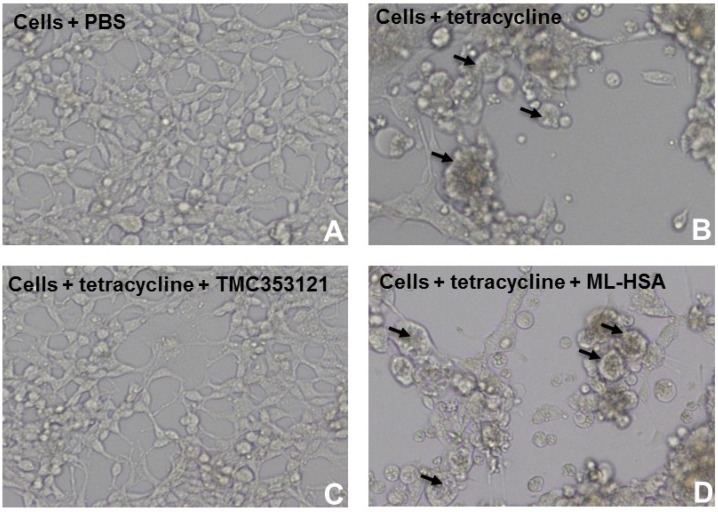
ML-HSA could not inhibit cell-cell fusion (or syncytium formation) mediated by F protein of RSV. 293-F cells expressing F protein of RSV induced by tetracycline was constructed. 293-F cells without tetracycline (**A**); 293-F cells with tetracycline (**B**), the arrows show the syncytia; 293-F cells with tetracycline in the presence of TMC353121 (200 nM), an F protein inhibitor (**C**); and 293-F cells with tetracycline in the presence of ML-HSA (1,000 nM) (**D**), and the arrows show the syncytia. Cell-cell fusion or syncytium formation was observed under a microscope.

To investigate whether ML-HSA could block the F protein-mediated membrane fusion, we performed a cell-cell fusion assay using 293 cells expressing F protein (293-F) induced with tetracycline. As shown in Figure 5, 293-F cells (Cell) did not form syncytia without tetracycline. After addition of tetracycline, syncytia were observable under a microscope. At the concentration of 1,000 nM (~100-fold higher than its IC_50_ for inhibiting RSV infection), ML-HSA and HSA did not inhibit the formation of syncytia. In contrast, TMC353121 at 200 nM significantly blocked syncytium formation (Figure 5).

### 3.4. ML-HSA Interacted with G Protein of RSV

**Figure 6:**
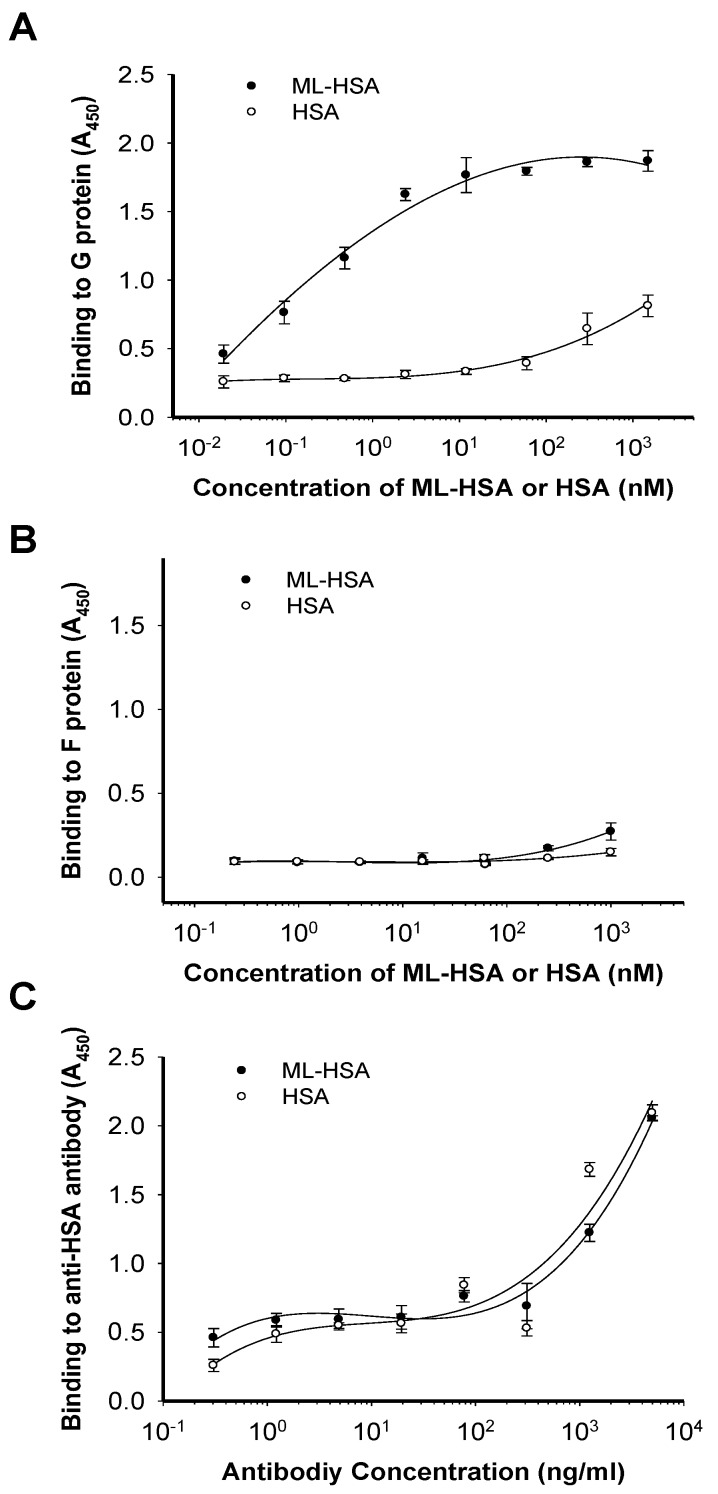
ML-HSA bound to RSV G protein. The wells of plates were coated with RSV G protein, HSA and ML-HSA, respectively. Binding of ML-HSA to RSV G protein (**A**), F protein (**B**) or anti-HSA antibody (**C**) was determined by ELISA. Each sample was tested in triplicate, and the experiment was repeated twice. The data from one representative experiment were presented in mean ± SD.

To determine whether ML-HSA blocks virus attachment to cells by interacting with RSV G protein, we performed an enzyme-linked immunosorbent assay (ELISA) using anti-HSA antibody to detect the binding of ML-HSA or HSA to RSV G protein and F protein, respectively, coated on the plate. The results showed that ML-HSA bound to G protein in a dose-dependent manner. Even at the concentration as low as 2.4 nM, ML-HSA still strongly bound to G protein (A_450_ = 1.62). HSA exhibited no significant binding to G protein at the concentration of 60 nM, although it showed weak binding at the concentration of 1,500 nM (Figure 6A). In contrast, both ML-HSA and HSA showed no significant binding to F protein at the concentration up to 1,000 nM (Figure 6B). To exclude the possibility that the difference of interaction with G protein between HSA and ML-HSA might result from the reduced reactivity of the chemically modified HSA to anti-HSA antibody, we performed another ELISA using anti-HSA antibody. As shown in Figure 6C, the anti-HSA antibody bound to both HSA and ML-HSA equally well, suggesting that ML-modified HSA maintains its antigenicity to react with anti-HSA antibody. These results confirm that ML-HSA blocks virus attachment to cells via its interaction with RSV G protein.

### 3.5. ML-HSA may Bind to the Middle Portion (Residues 145–186) of the G Protein Based on Drug-Resistance Study

**Figure 7:**
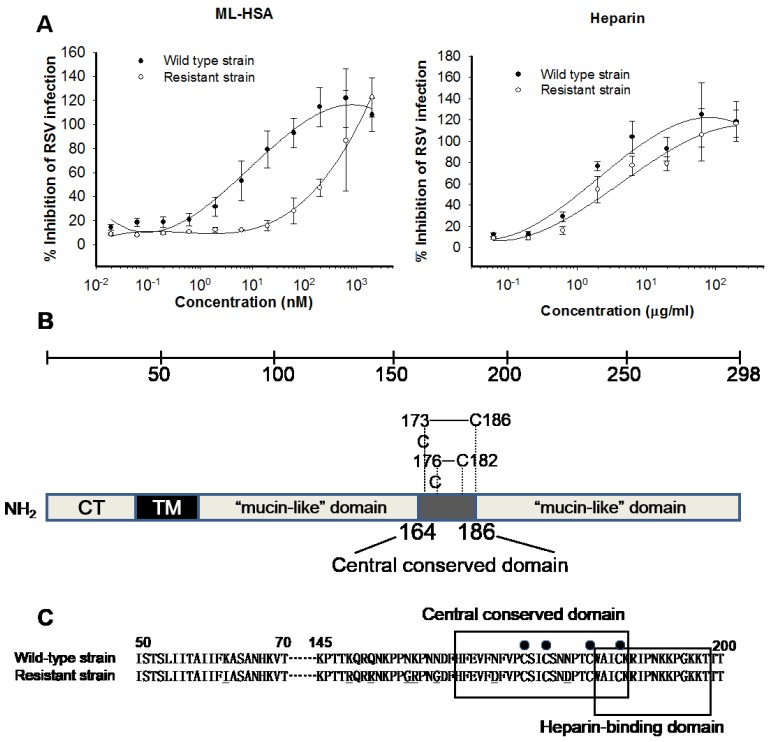
Determination of ML-HSA’s binding sites in RSV G protein by analysis of ML-HSA-induced resistant mutations. (**A**) Sensitivity of the RSV mutants and wild-type to ML-HSA (left panel) and heparin (right panel) was compared. Each sample was tested in triplicate, and the experiment was repeated twice. The data from one representative experiment were presented in mean ± SD. (**B**) Schematic presentation of the RSV Long Strain G protein and its functional domains. The G protein consists of a cytoplasmic domain (CT), a transmembrane domain (TM), two mucin-like domains, and a central conserved domain (residues 164–186). Four cysteine residues and the two corresponding disulfide bonds in the central conserved domain are indicated. (**C**) Amino acid sequences of the corresponding regions in the G protein of the RSV wild-type strain and ML-HSA-resistant strain. Bullets above the sequences indicate the four conserved cysteine residues. The central conserved domain and the heparin-binding domain are shown in boxes, respectively. The mutated amino acid residues in the G protein of ML-HSA-resistant strain are underlined.

To determine the binding site of ML-HSA in the RSV G protein, we selected a virus strain resistant to ML-HSA and compared its sensitivity to ML-HSA with that of the wild-type strain. As shown in Figure 7A, the wild-type strain showed high sensitivity to ML-HSA with an IC_50_ value of about 3.9 nM, while the resistant strain exhibited remarkable tolerance to ML-HSA, with an IC_50_ value of about 201 nM. Neither wild-type nor resistant strains showed resistance to heparin (Figure 7A). The viral RNA encoding G protein (Figure 7B) was sequenced and analyzed. The amino acid sequences of the G proteins of the wild-type strain (parent strain) and ML-HSA resistant strain were compared. We observed 8 amino acid mutations (K62I, K149R, Q152R, N157G, K158R, N161G, N169D, and N179D) in the G protein of the resistant strain, 7 of which were located in the region overlapping the residues 145–186, including the conserved domain (residues 164–186). These data suggest that ML-HSA may bind to the middle portion of the RSV G protein.

### 3.6. ML-HSA Inactivated RSV when RSV and ML-HSA Were Mixed before Intranasal Administration to Mice

**Figure 8:**
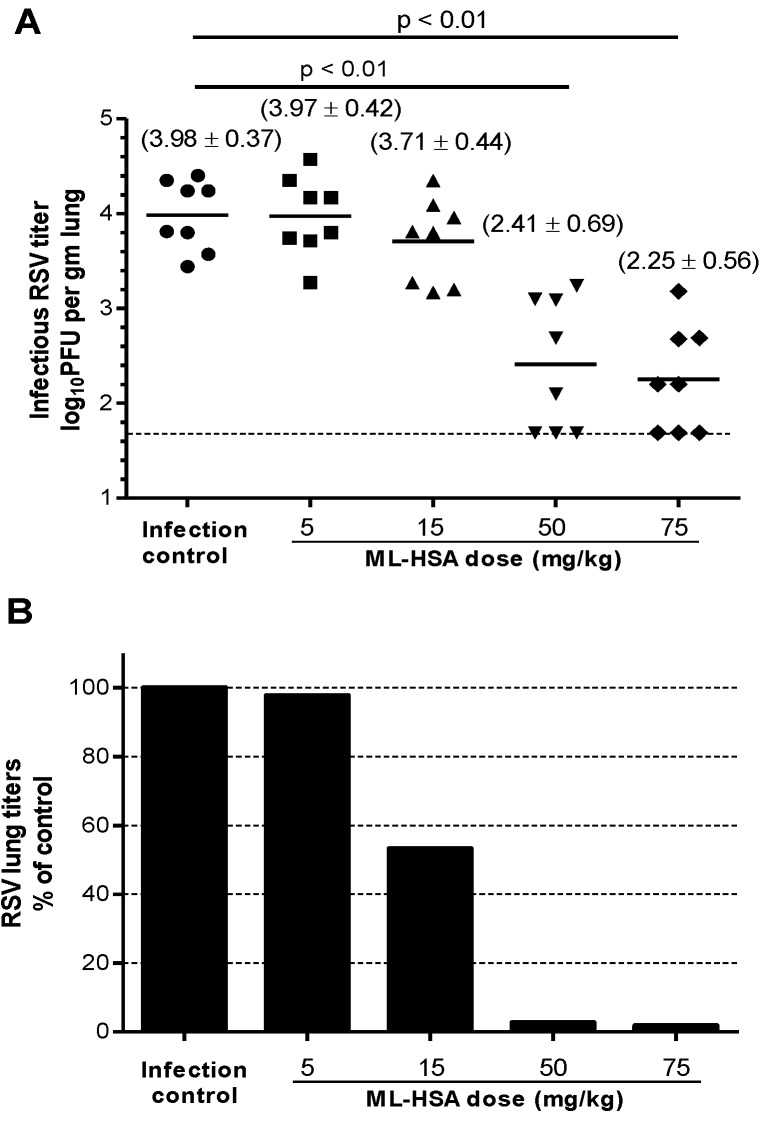
Inhibitory activity of ML-HSA on *in vivo* RSV infection in BALB/c mice. (**A**) ML-HSA at different concentrations was mixed with the RSV Long Strain before intranasal inoculation. The infectious RSV titer in the mouse lung was expressed as the log10 PFU per gram of lung tissue. Each data point represents the RSV titer for each individual animal in the respective treatment cohort. The horizontal line, drawn in each cohort, marks the geometric mean RSV titer of the group. The horizontal hatched line, across the graph, represents the RSV titer at the limit of assay detection. (**B**) Percentages of viral titer (geometric mean) in the lungs of ML-HSA-treated cohorts relative to infection control group (without ML-HSA treatment).

A mouse model of RSV infection was used to examine the *in vivo* inhibitory activity of ML-HSA. BALB/c mice (*n* = 8) were intranasally administered with ML-HSA at a single dose of 5, 15, 50 or 75 mg/kg, respectively, mixed with RSV Long Strain (2.1 × 10^5^ PFU). Four days post-infection, the mice were sacrificed, and viral titers in the lung homogenates were measured. As shown in Figure 8A, the viral titer in the untreated mice was in the range of 3.4 to 4.4 (mean = 3.98) log10 PFU per gram of lung tissue. The mean viral titers in the lungs of the mice treated with 5 and 15 mg/kg of ML-HSA were 3.97 and 3.7 log10 PFU per gram of lung tissue, respectively, thus showing no significant difference from the untreated group (*p* > 0.05). The mean viral titers in the lungs of mice treated with 50 and 75 mg/kg of ML-HSA were 2.41 and 2.25 log10 PFU per gram of lung tissue, respectively, which were significantly lower than those in the untreated control group (*p* < 0.01 for both groups). Thus, the RSV titer in the lungs of mice treated with 5, 15, 50, and 75 mg/kg of ML-HSA was reduced by 2.3%, 46.8%, 97.3% and 98.1%, respectively (Figure 8B), suggesting that ML-HSA, at the concentration of 50 and 75 mg/kg, can very effectively inhibit RSV replication *in vivo*.

### 3.7. ML-HSA Exhibited Prophylactic Efficacy in the Mouse Model against RSV Infection

**Figure 9:**
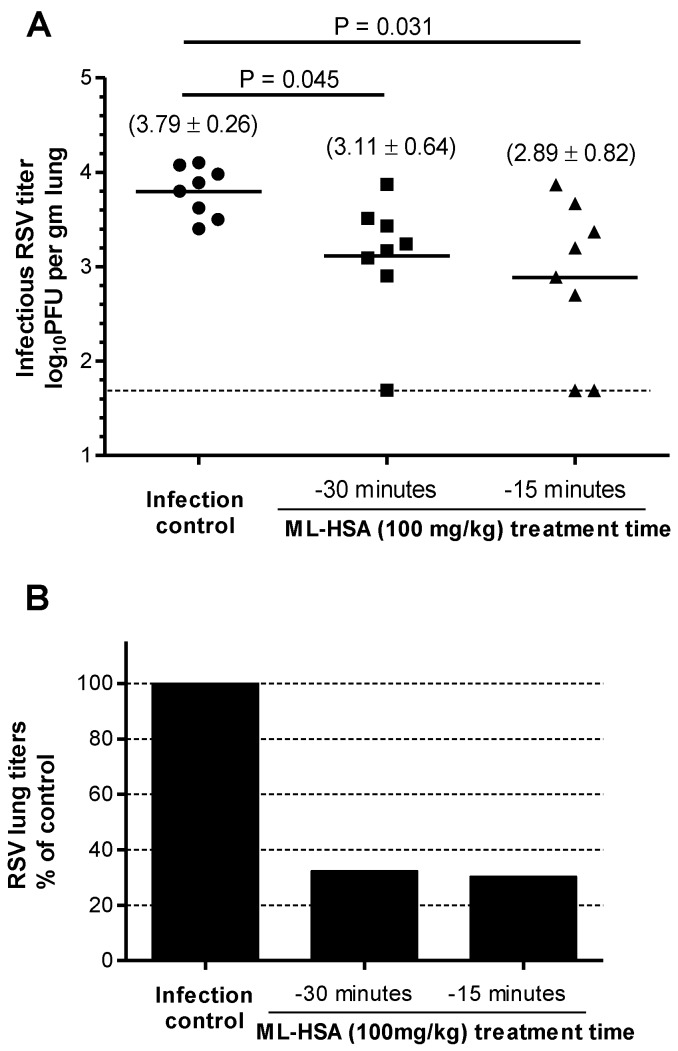
Prophylactic efficacy of ML-HSA against RSV infection in BALB/c mice. (**A**) ML-HSA (100 mg/kg) was administered 15 and 30 min, respectively, before RSV intranasal inoculation. The infectious RSV lung titer in the mouse lung was expressed as the log10 PFU per gram of lung tissue. Each data point represents the RSV titer for each individual animal in the respective treatment cohort. The horizontal line, drawn in each cohort, marks the geometric mean RSV titer of the group. The horizontal hatched line, across the graph, represents the RSV titer at the limit of assay detection. (**B**) Percentages of viral titer (geometric mean) in the lungs of ML-HSA-treated cohorts relative to infection control group (without ML-HSA treatment).

To evaluate the prophylactic efficacy of ML-HSA in the mouse model against RSV infection, we intranasally administered ML-HSA to mice at a dose of 100 mg/kg 30 or 15 min prior to RSV inoculation. Four days post-infection, PFU titers were determined using the plaque assay. The viral control cohort showed a mean of 3.79 log10 PFU per gram of lung tissue. The groups administered with ML-HSA 15 and 30 min, respectively, before RSV infection had a mean of 3.11 and 2.89 log10 PFU per gram of lung tissue, respectively (Figure 9A). The viral titers in the mice treated with ML-HSA 15 and 30 min, respectively, before RSV infection were significantly lower than those of the untreated mice (*p* = 0.045 and 0.031, respectively), with about 68% and 70% reduction of the viral titers in the lungs, respectively (Figure 9B). This result suggests that intranasal administration of ML-HSA is effective in preventing RSV infection.

## 4. Discussion

In the present study, we compared the *in vitro* anti-RSV activity of β-LG, BSA, OVA, and HSA modified with HP, ML and SU, respectively. While all these anhydride-modified proteins exhibited potent inhibitory activity against infection by RSV A2 and Long strains, HP- and ML-modified BSA and HSA were found to be more effective than other anhydride-modified proteins (Table 1). We selected ML-modified HSA for further study because (1) HSA is safer than BSA for human use, as some infants may be allergic to bovine proteins; and (2) the purchase price of HP is much higher than that of ML, making the production costs of ML-HSA much lower than those of HP-HSA. In addition, ML is a common anhydride used in pharmaceuticals. McCormick *et al.* have reported that maleic anhydride-divinyl ether copolymer (MVE-2) inhibits mammary and urinary bladder carcinogenesis [34]. Xie *et al*. demonstrated that FB006M, an anti-HIV-1 peptide modified with a ML derivative, exhibited no significant toxic effect to animals and human [35]. Based on our toxicity tests, ML-HSA showed no detectable cytotoxicity to HEp-2 and Vero cells (Figure 2).

HSA consists of 585 amino acids, including 59 lysine residues (10.1%) and 27 arginine residues (4.6%). Our previous studies have shown that modification of these positively charged amino acid residues converts the protein into a potent HIV entry inhibitor [36] and that the percentage of the modified lysine and arginine residues is dependent on the concentration of anhydrides and pH of the reaction system [13,19,34]. In this study, we found that the optimal pH value of system reaction was 9.0, while the optimal concentration of ML was 60 mM for preparation of ML-HSA with the most potent inhibitory activity against RSV infection (Figure 1A,B).

In the time-of-addition assay, ML-HSA exhibited significantly decreased inhibitory activity against RSV when it was added to the culture of cells and virus one hour post-viral infection (Figure 3), suggesting that ML-HSA inhibits RSV infection by targeting the early stages of viral replication. We then used a temperature shift assay to determine whether ML-HSA blocks viral attachment or binding to target cells based on the principle that RSV can attach or bind to target cells at 4 °C, while delaying cell fusion until the temperature is shifted to 18 °C or higher. Thus, if ML-HSA can block virus attachment to, but not fusion with the target cells, it will still retain inhibitory activity against RSV replication after the unbound ML-HSA and virus are removed following virus attachment at 4 °C, but before the temperature is raised to 18 °C or higher [33]. As shown in Figure 4, ML-HSA had similar IC_50_s whether or not when the unbound ML-HSA was removed before the temperature was shifted to 37 °C, confirming that ML-HSA blocks the attachment of RSV to the target cells, possibly by its interaction with the viral G protein. Indeed, ML-HSA binds with the G protein of RSV in a dose-dependent manner, as shown in ELISA (Figure 6A). In contrast, both ML-HSA and HSA showed no significant binding to F protein at the concentration up to 1000 nM (Figure 6B). However, our results, as herein presented, could not rule out the possibility that ML-HSA might also interact with F protein at a higher concentration in a non-specific way. In addition, the purified F protein that was tested here is likely in a post-fusion conformation, which is expected to be different from the pre-fusion conformation of RSV F protein. Therefore, further determining whether ML-HSA binds to the pre-fusion form of F protein is warranted.

The binding site of ML-HSA in viral G protein was revealed by induction of RSV mutants with resistance to ML-HSA. Most of the mutated residues are located in the region spanning residues 145–186, suggesting that ML-HSA may bind to the middle portion of the G protein. Interestingly, two Asn residues in this region were substituted with the negatively charged Asp residues (N169D and N179D), leading to increased net negative charges in the binding site of ML-HSA in the G protein, possibly resulting in the decreased electrostatic interaction between the negatively charged ML-HSA and G protein of RSV.

To the best of our knowledge, no RSV vaccine is currently available [37]. Palivizumab, an anti-RSV mAb, has been used as an immunoprophylaxis to prevent RSV infection in infants at high risk of RSV infection, including those with a history of prematurity, chronic lung disease, and congenital heart disease [38]. However, its high cost is prohibitive in low-income countries. As demonstrated in this study, ML-HSA is highly effective in inhibiting RSV infection *in vitro* in cell culture and *in vivo* in a mouse model. Furthermore, ML-HSA significantly reduced the titers of RSV in the lungs of mice when it was administered intranasally before challenge with RSV. Therefore, ML-HSA may be used as a prophylactic agent to prevent RSV infection in infants at high-risk or immunocompromised persons of RSV infection. However, unlike palivizumab, which must be intramuscularly injected by nurses or doctors in hospitals, clinics or doctors’ offices, ML-HSA can be intranasally administered by the parents of infants in the home or other places. Because of its intranasal application, ML-HSA may be approved for over-the-counter use. Palivizumab must be maintained and transported under low temperature, while ML-HSA may be kept at room temperature for at least 12 weeks based on its high stability (Figure 2C,D). Our previous study has also shown that the HP-modified LG protein maintained its antiviral activity for 3 months when it was kept at room temperature [12,38]. Therefore, ML-HSA might be made into a nasal spray or any other portable form for use with infants at high risk or immunocompromised persons before travelling to high-risk areas, such as hospitals or densely populated public areas, or used directly at such places to acquire protection against RSV cross-infection for a period. In our mouse model of RSV infection, ML-HSA administered 30 min prior to RSV infection yielded a significant protection. However, purified RSV with a high titer was used to infect BALB/c mice, differing from human infection with RSV virions which, in high-risk areas, are suspended in the air with low titer and, hence, easily spread. Therefore, in actual use, longer protection might be obtained with ML-HSA against RSV infection. While this topic outside the scope of the present work, further formulation development and clinical trials make this determination.

In conclusion, maleic anhydride-modified human serum albumin (ML-HSA) exhibited potent inhibitory activities against RSV infection *in vitro* and *in vivo* by binding to the viral G protein and blocking RSV attachment to the target cells. ML-HSA administered intranasally before RSV infection led to a significant reduction of viral titers in the lungs of mice. These results suggest that ML-HSA is a promising therapeutic candidate for further development into an effective, safe, and affordable intranasal regimen for pre-exposure prophylaxis of RSV infection in high-risk populations, including infants born prematurely with chronic lung or congenital heart disease, elderly people and immunocompromised adults.
